# Hypertension associated with serotonin reuptake inhibitors: A new analysis in the WHO pharmacovigilance database and examination of dose-dependency

**DOI:** 10.1371/journal.pone.0317841

**Published:** 2025-03-07

**Authors:** Basile Chrétien, Andry Rabiaza, Nishida Kazuki, Sophie Fedrizzi, Marion Sassier, Charles Dolladille, Joachim Alexandre, Xavier Humbert

**Affiliations:** 1 Center for Advanced Medicine and Clinical Research, Section of Biostatistics, Nagoya University Graduate School of Medicine, Aichi, Japan,; 2 Normandie Univ, UNICAEN, Department of general practice, Caen, France,; 3 CHU Caen, Pharmacology Department, Caen, France,; 4 Normandie Univ, UNICAEN, INSERM U1086 ANTICIPE, Caen, France; Cholistan University of Veterinary and Animal Sciences, PAKISTAN

## Abstract

**Introduction:**

Recent literature has reported instances of drug associated with hypertension with serotonin reuptake inhibitors (SRIs). Nonetheless, the association between SRIs and hypertension development is the subject of ongoing debate. It remains uncertain whether this is indicative of a class effect, and if dose-effect exist. To investigate the potential class effect associating SRIs with hypertension reporting, we utilized real-world data from VigiBase®, the World Health Organization (WHO) pharmacovigilance database.

**Methods:**

We conducted an updated disproportionality analysis within VigiBase® to identify a signal of hypertension reporting with individual SRIs by calculating adjusted reporting odds ratios (aRORs) within a multivariate case/non-case study design. Additionally, we explored the presence of a dose-effect relationship.

**Results:**

The database contained 13,682 reports of SRI associated with hypertension (2.2%), predominantly in women (70.0%). Hypertension was most reported in the 45-64 years old age group (44.8%). A total of 3,879 cases were associated with sertraline, 2,862 with fluoxetine, 2,516 with citalopram, 2,586 with escitalopram, 2,441 with paroxetine, 201 with fluvoxamine and 8 with zimeldine.

A significant ROR was observed for all SRIs in both univariate (RORs ranging from 1.39 to 1.54) and multivariable analyses (aRORs ranging from 1.16 to 1.40) after adjustments for age group, sex, concurrent antihypertensive medication and drugs knowns to induce hypertension, except for fluvoxamine and zimeldine. No dose-response relationship was identified.

**Conclusion:**

This investigation, conducted under real life conditions, unveils a notable pharmacovigilance safety signal associating SRI usage with hypertension reporting. No dose-response effect was detectable. Further longitudinal studies are warranted.

## Introduction

Hypertension significantly contributes as a risk factor for cardiovascular diseases (CVD) [1,2]. Although essential hypertension predominates clinically, secondary hypertension can emanate from conditions such as renal parenchymal disease, renal artery stenosis, hyperaldosteronism, or pheochromocytoma. Pharmacologically induced hypertension, though less acknowledged, can lead to induced or uncontrolled hypertension [3].

A case-control study involving 700 participants across two hospitals revealed that a history of depression or anxiety correlated with a heightened risk of uncontrolled hypertension (adjusted OR 1.82, 95% CI: 1.27-2.60) [4]. Furthermore, the conjecture that antidepressants could significantly influence blood pressure—either directly or by moderating depression—has emerged [5]. Monoamine oxidase inhibitors (MAOIs), for example, can precipitate severe hypertension in the presence of tyramine-rich food or amphetamine consumption through the potentiation of monoamines like norepinephrine [6]. Tricyclic antidepressants, in a cohort study of 2,981 subjects, manifested a greater likelihood of developing stage 1 hypertension (OR 1.90, 95% CI 0.94-3.84, p < 0.07) and stage 2 hypertension (OR 3.19, 95% CI 1.35-7.55, p < 0.01) [7]. However, tricyclic antidepressant only showed a trend toward grade 1 hypertension in this paper, as the threshold of statistical significance was not reached. These compounds could also induce hypertensive crises in patients with unrecognized pheochromocytoma [8,9]. Consequently, serotonin reuptake inhibitors (SRIs) are touted as first-line antidepressants, given their comparatively lower risk profile for adverse drug reactions (ADRs) [10]. Nonetheless, SRI-associated hypertension debate persists, and hypertension is not universally acknowledged as a possible adverse effect in the Summaries of Product Characteristics (SPC) of this drug class. Hence, following prior indications of a pharmacovigilance signal for SRI associated hypertension in VigiBase, we now conduct an updated investigation with enhanced statistical power and refined methodology accounting for potential confounders and seek to elucidate any dosage-dependent association. This is imperative for advancing patient care optimization [11].

To investigate the potential class effect associating SRIs with hypertension reporting, we utilized real-world data from VigiBase®, the World Health Organization (WHO) pharmacovigilance database and to determine if a dose-effect association was found with SRI- hypertension couples.

## Materials and methods

### Population/Data source

Our analyses harnessed VigiBase®, the WHO’s comprehensive Individual Case Safety Report (ICSR) database [12]. Encompassing more than 35 million reports from 120 nations since 1968, ICSRs integrate administrative data (reporting country, report type, reporter qualifications), patient demographics (sex, age), reaction onset date, outcome characterization using MedDRA version 27.1 terms, WHO causality assessment, and detailed drug information (name, administration and cessation dates, induction period, prescribed indication, dosage, dechallenge, rechallenge), accompanied by report completeness levels. Each ADR was classed as either ‘serious’ or ‘non-serious’ based on WHO criteria, with ‘serious’ encompassing outcomes such as death, life-threatening situations, hospitalization or its prolongation, persistent incapacity or disability, and clinically significant events as judged by the reporting physician [13].

### Case/Non-case study in VigiBase®

ICSRs with reported hypertension—based on MedDRA *System Organ Class version* 27.1’s “Hypertension (broad)” Standardized MedDRA Query (SMQ) definition—from 1968 to the 29^th^ of July 2024 comprised our ‘cases’ within the adult demographic, while ‘non-cases’ involved all adult reports devoid of hypertension. Terms included in the “Hypertension (broad)” (SMQ) are described in detail in Supplementary S3 Table. We classified drug exposure by detecting at least one SRI as per the Anatomical Therapeutic and Clinical classification (ATC) within a report. All SRI were included in the analysis regardless of their status (suspect, concomitant or interaction). The analysis of disproportionality in this case/non-case format, aimed to examine the association between drug exposure and hypertension occurrence [14–16], facilitated the estimation of reporting odds ratios (RORs) and their 95% confidence intervals (CIs) for each SRI.

### Statistical analysis

Data were expressed as means ±  SD or percentage. For the case/non-case analysis, we estimated the ROR with 95% CI for each SRI-hypertension pair, and a safety signal was identified with an ROR > 1 and a lower 95% CI limit > 1. Furthermore, multivariable analyses were performed, adjusting for age, sex, concurrent C02 ATC class antihypertensive reports, and drugs known to induced hypertension as defined by Foy et al. [17] to reduce bias using adjusted reporting odds ratio (aRORs). The investigation of dose-response relationships involved ROR computations for dosages less than or equal to the median and those exceeding it for each SRI, with a linear regression analysis conducted likewise. Time to onset, number of positives dechallenges and rechallenges were also described. A sensitivity analysis was done using only reports with suspected SRIs (according to the pharmacovigilance criteria). Another sensitivity analyses was done in a population of patients treated with antidepressants (defined by the N06A ATC class). The analyses encompassed the collective SRIs and adhered to the READUS-PV guidelines with an execution in R 4.4.1 [18,19].

## Results

### Descriptive characteristics

**Fig 1:**
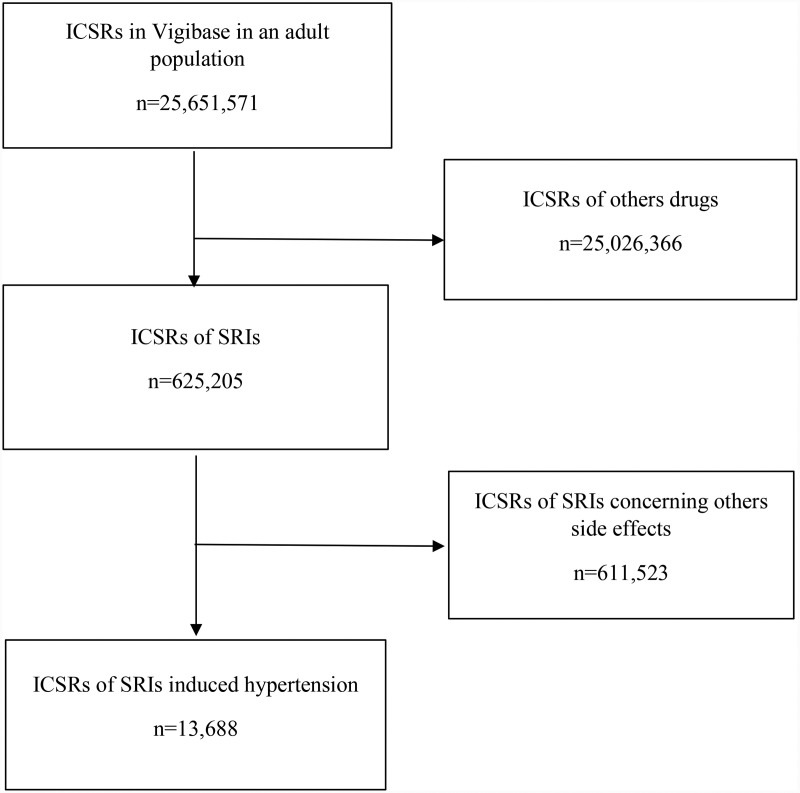
Flow chart of individual case safety report (ICSR) in WHO pharmacovigilance database concerning serotonin reuptake inhibitors (SRIs) induced hypertension.

VigiBase® reports pertinent to the included SRIs totaled 625,205. Adhering to the selection criteria, we identified 13,688 hypertensive cases (2.2%) (Fig 1).

SRI associated with hypertension predominately affected women (70.0%). Hypertension was most reported in the 45-64 years old age group (44.8%). Reports associated with sertraline numbered at 3,879; fluoxetine, 2,862; citalopram, 2,516; escitalopram, 2,586; paroxetine, 2,441; fluvoxamine, 201 and zimeldine, 8. Concomitant antihypertensive drug use was reported in 1,095 cases (8.0%), and a total of 1,752 reports were linked with CVDs (750 myocardial infarction cases and 1,002 stroke cases). Serious cases constituted 9,223 reports (75.5%) (Table 1).

**Table 1:** Characteristics of the 13,688 individual case safety reports (ICSRs) of hypertension with SRIs reported in VigiBase®.

Sex	N available	13,688
	Female	9,535 (70.0%)
Age	N available	13,688
	18-44	3,326 (24.3%)
	45-64	6,134 (44.8%)
	65-74	2,452 (17.9%)
	75 +	1,776 (13.0%)
Serious	N available	12,208
	Yes	9,223 (75.5%)
Seriousness criterias^*^	Caused/Prolonged Hospitalization	4,287 (46.5%)
	Congenital anomaly/Birth defect	0
	Death	709 (7.7%)
	Disabling/Incapacitating	247 (2.7%)
	Life threatening	523 (5.7%)
	Other	3,358 (36.4%)
	NA’s	4,564 (49.5%)
Concomitant stroke		1,002 (7.3%)
Concomitant myocardial infarction		750 (5.5%)
Concomitant antihypertensive drug		1,095 (8.0%)
Concomitant potentially hypertensive drug		5,575 (40.7%)

* : several seriousness criteria can be added at the same time.

Time to onset and positive dechallenge - rechallenge are described in Table 2 and 3 respectively.

**Table 2:** Time to onset (TTO) of hypertension after SRIs exposure.

Drug	Number of reports with available information	TTO in days (median, interquartile range)
Alaproclate	0	NA
Citalopram	157	2.0 (0.0-16.0)
Escitalopram	136	1.0 (0.0-12.5)
Etoperidone	0	NA
Fluoxetine	249	13.0 (1.0-66.0)
Fluvoxamine	59	9.0 (1.0-38.0)
Paroxetine	310	5.0 (1.0-119.0)
Sertraline	388	4.5 (0.0-56.0)
Zimeldine	5	1.0 (1.0-4.0)

**Table 3:** Dechallenge and rechallenge after the onset of hypertension.

Drug	Positive dechallenge	Positive rechallenge
Alaproclate	0	0
Citalopram	138	3
Escitalopram	168	2
Etoperidone	0	0
Fluoxetine	253	15
Fluvoxamine	41	2
Paroxetine	212	5
Sertraline	275	12
Zimeldine	1	0

### Univariate and multivariate case/non-case analysis in VigiBase

Table 4 illustrates that SRIs elicited a significant signal for hypertension in bivariate analysis (RORs ranged from 1.43 to 1.58) and multivariable analysis (aRORs ranged from 1.16 to 1.40) post-adjustment for age group, sex, concurrent antihypertensive medication and drugs knowns to induce hypertension, except for fluvoxamine and zimeldine.

**Table 4:** Disproportionality analysis with all SRIs in VigiBase® to search for a signal of hypertension.

Drug Name	Cases (=a)	Non-cases (=b)	Bivariate analysis		Multivariable analysis^*^	
			ROR	95%CI	ROR	95%CI
alaproclate	0	1	NA			
citalopram	2,516	113,000	1.43	(1.37-1.49)	1.16	(1.12-1.21)
escitalopram	2,586	109,160	1.52	(1.46-1.58)	1.25	(1.20-1.30)
etoperidone	0	34	NA			
fluoxetine	2,862	125,242	1.47	(1.41-1.52)	1.29	(1.25-1.34)
fluvoxamine	201	12,673	1.01	(0.88-1.17)	0.99	(0.86-1.14)
paroxetine	2,441	9,8904	1.58	(1.52-1.65)	1.40	(1.34-1.46)
sertraline	3,879	165,784	1.50	(1.45-1.55)	1.30	(1.26-1.35)
zimeldine	8	910	0.56	(0.28-1.13)	0.59	(0.29-1.18)
SRI class	13,688	610,679	1.45	(1.43-1.48)	1.33	(1.31-1.35)

* adjusted on age category, sex, antihypertensive drugs associated (ATC codes) and drugs known to induce hypertension.

### Dose-effect SRIs associated with hypertension

No dose-response relationship emerged (Table 5).

**Table 5:** Disproportionality analysis of the signal of hypertension associated with SRIs to search for a dose-dependency effect.

Drug Name	Doses ≤ to the median				Doses> to the median				P * ^2^
	Bivariate analysis		Multivariable analysis * ^1^		Bivariate analysis		Multivariable analysis * ^1^		
	ROR	95%CI	ROR	95%CI	ROR	95%CI	ROR	95%CI	
alaproclate	NA								
citalopram	1.81	(1.59-2.07)	1.16	(1.12-1.21)	2.02	(1.68-2.42)	1.70	(1.42-2.04)	0.70
escitalopram	1.40	(1.20-1.64)	1.25	(1.20-1.30)	1.6	(1.37-1.86)	1.47	(1.26-1.72)	0.71
etoperidone	NA								
fluoxetine	1.62	(1.38-1.90)	1.29	(1.25-1.34)	1.76	(1.45-2.14)	1.57	(1.29-1.90)	0.71
fluvoxamine	1.17	(0.58-2.35)	0.99	(0.86-1.14)	1.00	(0.59-1.69)	0.93	(0.54-1.61)	0.84
paroxetine	1.70	(1.45-1.98)	1.40	(1.34-1.46)	2.82	(2.35-3.39)	2.51	(2.09-3.01)	0.62
sertraline	1.99	(1.83-2.18)	1.30	(1.26-1.35)	0.92	(0.44-1.95)	0.83	(0.39-1.75)	0.58
zimeldine	NA				NA				

*^1^: adjusted on age category, sex, antihypertensive drugs associated (ATC codes) and drugs known to induce hypertension, * ^2^: linear regression model. Model was run only if at least 5 reports of hypertension were made for each drug in the conditions given in this table.

### Sensitivity analysis

Disproportionality analysis with all suspected SRIs in VigiBase® to search for a signal of hypertension found no pharmacovigilance signal except for sertraline [RORa = 1.07 (1.00;1.15)] in multivariate analyses (Supplementary S1 Table). In another hand, disproportionality analysis with SRIs in VigiBase® to search for a signal of hypertension in a population of patients treated with antidepressants found a pharmacovigilance signal for citalopram, escitalopram, fluoxetine, paroxetine and sertraline in multivariate analyses (Supplementary S2 Table).

## Discussion

This investigation substantiates a pharmacovigilance signal for hypertension in patients administered SRIs, despite adjustments for multiple confounders. A class effect seems plausible, yet no dose-dependency was identified. Past VigiBase studies on this topic are sparse, potentially with suboptimal methodologies. Our prior work relied solely on univariate analysis, neglected potential confounders, and omitted the search for dose-dependency relationships [11]. Montastruc et al. identified a positive correlation between the Norepinephrine Transporter/Serotonin transporter pKi ratio and hypertension reports associated with SRI and SNRI antidepressants using univariate analysis [20].

Controversy surrounds SRI associated with hypertension, with only sertraline and paroxetine SPCs explicitly noting hypertension risk. These two agents, in pre-clinical and phases II-III trials, were connected to potential hypertension manifestations [21–23], unlike the other four agents [24–29], which may account for this inconsistency. Regrettably, these studies predominantly featured normotensive subjects, which do not perfectly mirror real-life patient treatments. Elsewhere, Chen et al. have realized an evaluation of the association between the six most commonly prescribed SRIs and CVD adverse events, using the FDA Adverse Event Reporting System (FAERS) from Q1 2004 to Q2 2022. Their disproportionality analysis showed a significant association between SRIs and the CVD adverse events (arrhythmias, *torsades de pointes*/QT prolongation, cardiomyopathy, and hypertension), with higher signals in middle-aged and elderly patients and women [30]. However, Zhang et al. have previously shown that SRIs help to reduce peak blood pressure in hypertensive patients with depression in a metanalysis including only six studies, and 149 patients with CVD and depression. Potential pathophysiological mechanisms may lie in serotonin function, sympathetic activation and genetic heterogeneity [31].

Hypertension mechanisms induced by SRIs might involve the blockade of serotonin transporters, causing increased extracellular monoaminergic neurotransmitter concentrations [32]. In vivo and in vitro evidence suggests that heightened serotonin levels can cause arterial vasoconstriction [33,34] and that SRIs alter serotonin uptake in platelets [35]. Other studies have speculated that SRIs might hamper nitric oxide synthesis, a key vasodilator integral to vascular tone and reactivity [36]. Katsi et al.‘s narrative review in 2013 found minimal SRI effects on blood pressure [37]. However, Grossman et al. highlighted fluoxetine associated with hypertension in case studies [38]. Amsterdam et al. observed a marginal incidence of sustained hypertension (1.7%) during short-term fluoxetine treatment at a daily dosage of 20mg over up to 12 weeks in 796 depressed patients [39]. Experimental investigations also demonstrated fluoxetine’s potential to raise blood pressure under various conditions (e.g., in ambulatory rats, dogs with neurogenic orthostatic hypotension, and individuals with idiopathic Parkinson’s disease) [40–42]. Montastruc et al. noted a mild yet clinically significant impact of fluoxetine on hemodynamic factors, including blood pressure, in parkinsonian patients with orthostatic hypotension [41]. From a pathophysiological point of view, these same authors have suggested that the NET/SERT ratio could be involved, although they tend to favour the determining role of NET over the serotonergic effect [20]. Furthermore, an orthostatic hypotension-simulating tilt test in chronically sino-aortic denervated dogs under chloralose anaesthesia yielded analogous results [40]. Lazartigues et al. discovered that acute central fluoxetine administration induced a pressor response via enhancement of sympathetic tone and vasopressin secretion, hinting at the potential applicability of α_1_ -adrenoceptor and/or V_1A_ -vasopressin receptor antagonists in managing ‘Serotonin Syndrome’ [42]. Research on hypertension induced by other SRIs remains scant.

Highlighted strengths of this study include the utilization of the validated case/non-case methodology for ADR signal detection in pharmacovigilance data [15,16]. VigiBase®, with its global report aggregation, offers diverse medical practices and patient characteristics. The enhanced methodology, featuring multivariable analysis, confers greater relevance to this latest analysis. Notwithstanding, limitations common to pharmacovigilance database investigations persist, such as the prominence of under-reporting, which, while important, does not detract from the validity and significance of the case/non-case methodology [15]. Missing data also represents a limitation in pharmacovigilance database extractions. Additionally, given depression’s potential impact on blood pressure, discerning the precise influence of SRIs on blood pressure remains challenging. However, given the absence of signal of hypertension in the analysis including only suspected SRIs, we cannot exclude with certainty that the signal found in our main analysis could be driven by unidentified confounding factors. It is indeed possible that a part of our signal is driven by the fact that depressive symptoms are associated with a greater risk of developing hypertension [43]. Furthermore, the analysis comparing SRIs to other antidepressants indicates that SRIs were associated with a lower signal for hypertension. However, this finding might be influenced by a notoriety bias favoring these antidepressants, which ultimately makes our hypothesis plausible. The short time to onset of hypertension after SRIs initiation, as well as the number of positive dechallenges and rechallenges are also in favour of our hypothesis.

## Conclusions

This updated analysis, carried out under real-world circumstances, has disclosed a significant pharmacovigilance safety signal linking SRI administration with the onset or exacerbation of hypertension. From a clinical perspective, this study implies that physicians treating patients with depressive disorders must recognize the potential for SRIs to elevate blood pressure akin to other antidepressants. Consequently, routine blood pressure monitoring is advisable for these patients. Given the absence of a dose-effect relationship, clinicians should determine SRI dosing based on symptomatology and observed guidelines.

## Supporting information

S1 Table Disproportionality analysis with all suspected SRIs in VigiBase® to search for a signal of hypertension. * adjusted on age category, sex, antihypertensive drugs associated (ATC codes) and associated drugs known to induce hypertension (ATC codes).
(DOCX)

S2 Table Disproportionality analysis with SRIs in VigiBase® to search for a signal of hypertension in a population of patients treated with antidepressants. * adjusted on age category, sex, antihypertensive drugs associated (ATC codes) and associated drugs know to induce hypertension (ATC codes). Note: the population was defined using N06A ATC codes (antidepressant), and not with International Non Proprietary Names, which explains the slight difference in the number of cases between this sensitivity analyses and the main one.
(DOCX)

S3 Table Terms included in the SMQ “Hypertension (broad)” definition based on MedDRA SOC. (DOCX)

## Abbreviations:

ADR: Adverse Drug Reaction
aROR: adjusted Reporting Odds Ratio
ATC: Anatomical Therapeutic and Clinical classification
CVD: Cardiovascular Disease
FAERS: FDA Adverse Event Reporting System
ICSR: Individual Case Safety Report
MAOI: Monoamine Oxidase Inhibitors
ROR: Reporting Odds Ratio
SMQ: Standardized MedDRA Query
SPC: Summary of Product Characteristics
SRI: Serotonin Reuptake Inhibitor
WHO: World Health Organization.
CI: Confidence Interval

## References

[1] Stokes J 3rd, Kannel WB, Wolf PA, D’Agostino RB, Cupples LA. Blood pressure as a risk factor for cardiovascular disease. The Framingham Study--30 years of follow-up. Hypertension. 1989;13(5 Suppl):I13–8. doi: 10.1161/01.hyp.13.5_suppl.i13 2535213

[2] Flack JM, Neaton J, Grimm R Jr, Shih J, Cutler J, Ensrud K, et al. Blood pressure and mortality among men with prior myocardial infarction. Multiple Risk Factor Intervention Trial Research Group. Circulation. 1995;92(9):2437–45. doi: 10.1161/01.cir.92.9.2437 7586343

[3] Grossman E, Messerli FH. Drug-induced hypertension: an unappreciated cause of secondary hypertension. Am J Med. 2012;125(1):14–22. doi: 10.1016/j.amjmed.2011.05.024 22195528

[4] Almas A, Patel J, Ghori U, Ali A, Edhi AI, Khan MA. Depression is linked to uncontrolled hypertension: a case-control study from Karachi, Pakistan. J Ment Health. 2014;23(6):292–6. doi: 10.3109/09638237.2014.924047 24949520

[5] Fu L, Ma L, Zhao X, Li Y, Zhu H, Yang W. Antidepressant medication can improve hypertension in elderly patients with depression. J Clin Neurosci. 2015;22:1911–5. 26256065 10.1016/j.jocn.2015.03.067

[6] Rossi GP, Seccia TM, Maniero C, Pessina AC. Drug-related hypertension and resistance to antihypertensive treatment: a call for action. J Hypertens. 2011;29(12):2295–309. doi: 10.1097/HJH.0b013e32834c465d 22002334

[7] Licht CMM, de Geus EJC, Seldenrijk A, van Hout HPJ, Zitman FG, van Dyck R, et al. Depression is associated with decreased blood pressure, but antidepressant use increases the risk for hypertension. Hypertension. 2009;53(4):631–8. doi: 10.1161/HYPERTENSIONAHA.108.126698 19237679

[8] Ferguson KL. Imipramine-provoked paradoxical pheochromocytoma crisis: a case of cardiogenic shock. Am J Emerg Med. 1994;12(2):190–2. doi: 10.1016/0735-6757(94)90245-3 8161395

[9] Korzets A, Floro S, Ori Y, Weizer N, Gruzman C. Clomipramine-induced pheochromocytoma crisis: a near fatal complication of a tricyclic antidepressant. J Clin Psychopharmacol. 1997;17(5):428–30. doi: 10.1097/00004714-199710000-00019 9315999

[10] Anderson IM, Nutt DJ, Deakin JF. Evidence-based guidelines for treating depressive disorders with antidepressants: a revision of the 1993 British Association for Psychopharmacology guidelines. British Association for Psychopharmacology. J Psychopharmacol. 2000;14(1):3–20. doi: 10.1177/026988110001400101 10757248

[11] Humbert X, Fedrizzi S, Chrétien B, Sassier M, Bagheri H, Combret S, et al. Hypertension induced by serotonin reuptake inhibitors: analysis of two pharmacovigilance databases. Fundam Clin Pharmacol. 2019;33(3):296–302. doi: 10.1111/fcp.12440 30489655

[12] Bate A, Lindquist M, Edwards I. The application of knowledge discovery in databases to post-marketing drug safety: example of the WHO database. Fundamental & Clinical Pharmacology. 2008;22 127–40. 18248442 10.1111/j.1472-8206.2007.00552.x

[13] Edwards IR, Aronson JK. Adverse drug reactions: definitions, diagnosis, and management. Lancet. 2000;356(9237):1255–9. doi: 10.1016/S0140-6736(00)02799-9 11072960

[14] Egberts ACG, Meyboom RHB, van Puijenbroek EP. Use of measures of disproportionality in pharmacovigilance: three Dutch examples. Drug Saf. 2002;25(6):453–8. doi: 10.2165/00002018-200225060-00010 12071783

[15] Montastruc J-L, Sommet A, Bagheri H, Lapeyre-Mestre M. Benefits and strengths of the disproportionality analysis for identification of adverse drug reactions in a pharmacovigilance database. Br J Clin Pharmacol. 2011;72(6):905–8. doi: 10.1111/j.1365-2125.2011.04037.x 21658092 PMC3244636

[16] Faillie J-L. Case-non-case studies: Principle, methods, bias and interpretation. Therapie. 2019;74(2):225–32. doi: 10.1016/j.therap.2019.01.006 30773344

[17] Foy MC, Vaishnav J, Sperati CJ. Drug-Induced Hypertension. Endocrinol Metab Clin North Am. 2019;48(4):859–73. doi: 10.1016/j.ecl.2019.08.013 31655781

[18] Fusaroli M, Salvo F, Begaud B, AlShammari TM, Bate A, Battini V, et al. The REporting of A Disproportionality Analysis for DrUg Safety Signal Detection Using Individual Case Safety Reports in PharmacoVigilance (READUS-PV): Explanation and Elaboration. *Drug Saf* Published Online First: 7 May 2024. doi: 10.1007/s40264-024-01423-7 PMC11116264 38713347

[19] Fusaroli M, Salvo F, Begaud B, AlShammari TM, Bate A, Battini V, et al. The reporting of a disproportionality analysis for drug safety signal detection using individual case safety Reports in PharmacoVigilance (READUS-PV): Development and Statement. Drug Saf. 2024;47(6):575–84. doi: 10.1007/s40264-024-01421-9 38713346 PMC11116242

[20] Montastruc J-L, Rousseau V, de Canecaude C, Roussin A, Montastruc F. Role of serotonin and norepinephrine transporters in antidepressant-induced arterial hypertension: a pharmacoepidemiological-pharmacodynamic study. Eur J Clin Pharmacol. 2020;76(9):1321–7. doi: 10.1007/s00228-020-02913-0 32483650

[21] Mattila MJ, Saarialho-Kere U, Mattila M. Acute effects of sertraline, amitriptyline, and placebo on the psychomotor performance of healthy subjects over 50 years of age. J Clin Psychiatry. 1988;49 Suppl52–8. 3045113

[22] Lund J, Thayssen P, Mengel H, Pedersen OL, Kristensen CB, Gram LF. Paroxetine: pharmacokinetics and cardiovascular effects after oral and intravenous single doses in man. Acta Pharmacol Toxicol (Copenh). 1982;51(4):351–7. doi: 10.1111/j.1600-0773.1982.tb01036.x 6217723

[23] Yokota S, Ishikura Y, Ono H. Cardiovascular effects of paroxetine, a newly developed antidepressant, in anesthetized dogs in comparison with those of imipramine, amitriptyline and clomipramine. Jpn J Pharmacol 1987; 45:335–42. 2963926 10.1254/jjp.45.335

[24] Pedersen OL, Kragh-Sørensen P, Bjerre M, Overø KF, Gram LF. Citalopram, a selective serotonin reuptake inhibitor: clinical antidepressive and long-term effect--a phase II study. Psychopharmacology (Berl). 1982;77(3):199–204. doi: 10.1007/BF00464566 6812140

[25] Stein DJ, Andersen EW, Tonnoir B, Fineberg N. Escitalopram in obsessive-compulsive disorder: a randomized, placebo-controlled, paroxetine-referenced, fixed-dose, 24-week study. Curr Med Res Opin. 2007;23(4):701–11. doi: 10.1185/030079907x178838 17407626

[26] Fuller RW, Holland DR, Yen TT, Bemis KG, Stamm NB. Antihypertensive effects of fluoxetine and L-5-hydroxytryptophan in rats. Life Sci. 1979;25(14):1237–42. doi: 10.1016/0024-3205(79)90466-1 316092

[27] Visser M, Seidell JC, Koppeschaar HP, Smits P. No specific effect of fluoxetine treatment on fasting glucose, insulin, lipid levels, and blood pressure in healthy men with abdominal obesity. Obes Res. 1994;2(2):152–9. doi: 10.1002/j.1550-8528.1994.tb00641.x 16353616

[28] De Wilde JE, Doogan DP. Fluvoxamine and chlorimipramine in endogenous depression. J Affect Disord. 1982;4(3):249–59. doi: 10.1016/0165-0327(82)90009-x 6215443

[29] Wilson WH, Higano H, Papadatos Y, Kelwala S, Ban TA. A double-blind placebo-controlled study to compare the autonomic effects of fluvoxamine with those of amitriptyline and doxepin in healthy volunteers. Br J Clin Pharmacol. 1983;15 Suppl 3(Suppl 3):385S-392S. doi: 10.1111/j.1365-2125.1983.tb02129.x 6407500 PMC1427650

[30] Chen Y, Fan Q, Liu Y, Shi Y, Luo H. Cardiovascular toxicity induced by SSRIs: Analysis of spontaneous reports submitted to FAERS. Psychiatry Res. 2023;326 115300. doi: 10.1016/j.psychres.2023.115300 37364503

[31] Zhang L, Li G, Liu M. A meta-analysis on the association between SSRIs and blood pressure in patients with CVD and depression. J Affect Disord. 2023;340 181–8. doi: 10.1016/j.jad.2023.08.032 37557986

[32] De Vera MA, Bérard A. Antidepressant use during pregnancy and the risk of pregnancy-induced hypertension. Br J Clin Pharmacol. 2012;74(2):362–9. doi: 10.1111/j.1365-2125.2012.04196.x 22435711 PMC3630756

[33] Morrison JL, Chien C, Riggs KW, Gruber N, Rurak D. Effect of maternal fluoxetine administration on uterine blood flow, fetal blood gas status, and growth. Pediatr Res. 2002;51(4):433–42. doi: 10.1203/00006450-200204000-00007 11919327

[34] Watts S, Morrison S, Davis R, Barman S. Serotonin and blood pressure regulation. Pharmacological Reviews. 2012;64 359–88. 22407614 10.1124/pr.111.004697 PMC3310484

[35] Skop BP, Brown TM. Potential vascular and bleeding complications of treatment with selective serotonin reuptake inhibitors. Psychosomatics. 1996;37(1):12–6. doi: 10.1016/S0033-3182(96)71592-X 8600488

[36] Finkel MS, Laghrissi-Thode F, Pollock BG, Rong J. Paroxetine is a novel nitric oxide synthase inhibitor. Psychopharmacol Bull. 1996;32(4):653–8. 8993087

[37] Katsi VK, Marketou M, Vamvakou G, Makris T, Tousoulis D, Stefanadis CI, et al. Novel antidepressant drugs, arterial hypertension and cardiovascular disease. Recent Pat Cardiovasc Drug Discov. 2013;8(3):178–85. doi: 10.2174/1574890109666140407154738 24712681

[38] Grossman A, Messerli FH, Grossman E. Drug induced hypertension--An unappreciated cause of secondary hypertension. Eur J Pharmacol. 2015;763(Pt A):15–22. doi: 10.1016/j.ejphar.2015.06.027 26096556

[39] Amsterdam JD, Garcia-Espana F, Fawcett J, Quitkin FM, Reimherr FW, Rosenbaum JF, et al. Blood pressure changes during short-term fluoxetine treatment. J Clin Psychopharmacol. 1999;19(1):9–14. doi: 10.1097/00004714-199902000-00004 9934937

[40] Verwaerde P, Senard JM, Mazerolles M, Tran MA, Damase-Michel C, Montastruc JL, et al. Spectral analysis of blood pressure and heart rate, catecholamine and neuropeptide Y plasma levels in a new model of neurogenic orthostatic hypotension in dog. Clin Auton Res. 1996;6(2):75–82. doi: 10.1007/BF02291227 8726091

[41] Montastruc JL, Pelat M, Verwaerde P, Brefel-Courbon C, Tran MA, Blin O, et al. Fluoxetine in orthostatic hypotension of Parkinson’s disease: a clinical and experimental pilot study. Fundam Clin Pharmacol. 1998;12(4):398–402. doi: 10.1111/j.1472-8206.1998.tb00963.x 9711461

[42] Lazartigues E, Brefel-Courbon C, Bagheri H, Costes S, Gharib C, Tran MA, et al. Fluoxetine-induced pressor response in freely moving rats: a role for vasopressin and sympathetic tone. Fundam Clin Pharmacol. 2000;14(5):443–51. doi: 10.1111/j.1472-8206.2000.tb00426.x 11129084

[43] Inoue T. Depressive symptoms and the development of hypertension. Hypertens Res. 2024;47(11):3070–2. doi: 10.1038/s41440-024-01856-8 39169152

