# Supplementary material for: Hypertension associated with serotonin reuptake inhibitors: A new analysis in the WHO pharmacovigilance database and examination of dose-dependency
Source: PLoS One. 2025 Mar 7;20(3):e0317841. doi: 10.1371/journal.pone.0317841 (PMC11888134; doi:10.1371/journal.pone.0317841)
Supplement: S1 Table — * adjusted on age category, sex, antihypertensive drugs associated (ATC codes) and associated drugs known to induce hypertension (ATC codes). (DOCX) [file pone.0317841.s001.docx]

**S1 Table. Disproportionality analysis with all suspected SRIs in VigiBase® to search for a signal of hypertension.**

| Drug Name | Cases (=a) | Non-cases (=b) | Bivariate analysis | | Multivariable analysis* | |
| --- | --- | --- | --- | --- | --- | --- |
|  |  |  | ROR | 95%CI | ROR | 95%CI |
| alaproclate | 0 | 0 | NA | | | |
| citalopram | 446 | 30,332 | 0.94 | (0.86-1.03) | 0.94 | (0.86-1.04) |
| escitalopram | 471 | 31,901 | 0.94 | (0.86-1.03) | 0.97 | (0.89-1.07) |
| etoperidone | 0 | 18 | NA | | | |
| fluoxetine | 750 | 52,873 | 0.91 | (0.84-0.98) | 0.98 | (0.91-1.05) |
| fluvoxamine | 92 | 7,458 | 0.79 | (0.64-0.97) | 0.86 | (0.70-1.06) |
| paroxetine | 670 | 46,775 | 0.92 | (0.85-0.99) | 0.98 | (0.90-1.05) |
| sertraline | 862 | 54,323 | 1.02 | (0.95-1.09) | 1.07 | (1.00-1.15) |
| zimeldine | 8 | 890 | 0.58 | (0.29-1.15) | 0.61 | (0.30-1.21) |
| SRI class | 3,222 | 221,328 | 0.93 | (0.90-0.96) | 1.01 | (0.98-1.05) |

*adjusted on age category, sex, antihypertensive drugs associated (ATC codes) and associated drugs known to induce hypertension (ATC codes).
