# Supplementary material for: Hypertension associated with serotonin reuptake inhibitors: A new analysis in the WHO pharmacovigilance database and examination of dose-dependency
Source: PLoS One. 2025 Mar 7;20(3):e0317841. doi: 10.1371/journal.pone.0317841 (PMC11888134; doi:10.1371/journal.pone.0317841)
Supplement: S2 Table — * adjusted on age category, sex, antihypertensive drugs associated (ATC codes) and associated drugs know to induce hypertension (ATC codes). Note: the population was defined using N06A ATC codes (antidepressant), and not with International Non Proprietary Names, which explains the slight difference in the number of cases between this sensitivity analyses and the main one. (DOCX) [file pone.0317841.s002.docx]

**S2 Table. Disproportionality analysis with SRIs in VigiBase® to search for a signal of hypertension in a population of patients treated with antidepressants.**

| Drug Name | Cases (=a) | Non-cases (=b) | Bivariate analysis | | Multivariable analysis* | |
| --- | --- | --- | --- | --- | --- | --- |
|  |  |  | ROR | 95%CI | ROR | 95%CI |
| alaproclate | 0 | 1 | NA | | | |
| citalopram | 2,516 | 113,000 | 0.89 | (0.85-0.93) | 1.08 | (1.03-1.13) |
| escitalopram | 2,579 | 108,610 | 0.96 | (0.92-1.00) | 1.16 | (1.11-1.21) |
| etoperidone | 0 | 34 | NA | | | |
| fluoxetine | 2,846 | 124,677 | 0.91 | (0.88-0.95) | 1.16 | (1.11-1.21) |
| fluvoxamine | 201 | 12,673 | 0.64 | (0.55-0.73) | 0.88 | (0.77-1.02) |
| paroxetine | 2,436 | 98,584 | 1 | (0.96-1.04) | 1.28 | (1.22-1.34) |
| sertraline | 3,879 | 165,772 | 0.94 | (0.91-0.97) | 1.19 | (1.14-1.23) |
| zimeldine | 8 | 910 | 0.35 | (0.18-0.71) | 0.52 | (0.26-1.04) |
| SRI class | 13,654 | 610,077 | 0.83 | (0.81-0.85) | 0.87 | (0.85-0.89) |

*adjusted on age category, sex, antihypertensive drugs associated (ATC codes) and associated drugs know to induce hypertension (ATC codes).

Note : the population was defined using N06A ATC codes (antidepressant), and not with International Non Proprietary Names, which explains the slight difference in the number of cases between this sensitivity analyses and the main one.
