# Supplementary material for: Hypertension associated with serotonin reuptake inhibitors: A new analysis in the WHO pharmacovigilance database and examination of dose-dependency
Source: PLoS One. 2025 Mar 7;20(3):e0317841. doi: 10.1371/journal.pone.0317841 (PMC11888134; doi:10.1371/journal.pone.0317841)
Supplement: S3 Table — (DOCX) [file pone.0317841.s003.docx]

**S3 Table. Terms included in the SMQ “Hypertension (broad)” definition based on MedDRA SOC.**

| Accelerated hypertension |
| --- |
| Aldosterone high |
| Aldosterone increased |
| Aldosterone urine abnormal NOS |
| Aldosterone urine high |
| Aldosterone urine increased |
| Aldosteronism |
| Aldosteronism NOS |
| Aldosteronism secondary |
| Angiotensin I abnormal |
| Angiotensin I high |
| Angiotensin I increased |
| Angiotensin II abnormal |
| Angiotensin II high |
| Angiotensin II increased |
| Arterial pressure high |
| Arterial pressure NOS increased |
| Benign essential hypertension |
| Benign essential hypertension antepartum |
| Benign essential hypertension comp preg, childbirth, and the puerperium, unspec as to eoc |
| Benign essential hypertension complicating pregnancy, childbirth, and the puerperium |
| Benign essential hypertension complicating pregnancy, childbirth, and the puerperium, unspecified as |
| Benign essential hypertension with delivery |
| Benign essential hypertension, postpartum |
| Benign essential hypertension, with delivery, with mention of postpartum complication |
| Benign hypertensive heart disease |
| Benign hypertensive heart disease with congestive heart failure |
| Benign hypertensive heart disease without congestive heart failure |
| Benign renovascular hypertension |
| Benign secondary hypertension |
| Blood aldosterone abnormal |
| Blood aldosterone increased |
| Blood catecholamines abnormal |
| Blood catecholamines increased |
| Blood pressure abnormal |
| Blood pressure ambulatory abnormal |
| Blood pressure ambulatory increased |
| Blood pressure diastolic abnormal |
| Blood pressure diastolic high |
| Blood pressure diastolic increased |
| Blood pressure fluctuation |
| Blood pressure high |
| Blood pressure increased |
| Blood pressure increased refractory |
| Blood pressure labile |
| Blood pressure raised |
| Blood pressure systolic abnormal |
| Blood pressure systolic high |
| Blood pressure systolic increased |
| Blood renin activity high |
| Blood renin activity increased |
| Blood renin increased |
| BP raised |
| Catecholamines abnormal |
| Catecholamines elevated |
| Catecholamines increased |
| Catecholamines NOS abnormal |
| Catecholamines NOS high |
| Catecholamines NOS increased |
| Catecholamines NOS urine abnormal |
| Catecholamines NOS urine high |
| Catecholamines NOS urine increased |
| Catecholamines total abnormal NOS |
| Catecholamines urinary elevated |
| Change in blood pressure |
| Conn's syndrome |
| Crisis hypertensive |
| DBP increased |
| Diastolic blood pressure increased |
| Diastolic BP increased |
| Diastolic hypertension |
| Diastolic pressure increased |
| Eclampsia |
| Eclampsia complicating pregnancy, childbirth or the puerperium |
| Eclampsia complicating pregnancy, childbirth or the puerperium, unspecified as to episode of care |
| Eclampsia, antepartum |
| Eclampsia, postpartum |
| Eclampsia, with delivery |
| Eclampsia, with delivery, with mention of postpartum complication |
| Ectopic aldosterone secretion |
| Ectopic renin secretion |
| Elevated blood pressure reading without diagnosis of hypertension |
| Elevated BP |
| Encephalopathy hypertensive |
| Epinephrine abnormal NOS |
| Epinephrine high |
| Epinephrine increased |
| Essential hypertension |
| Essential hypertension, benign |
| Essential hypertension, malignant |
| Essential hypertension, unspecified |
| Grade 1 hypertensive fundus |
| Grade 2 hypertensive fundus |
| Grade 3 hypertensive fundus |
| Grade 4 hypertensive fundus |
| HBP |
| HT |
| Hyperaldosteronism |
| Hypertension |
| Hypertension aggravated |
| Hypertension arterial |
| Hypertension complicating pregnancy, childbirth, and the puerperium |
| Hypertension diastolic |
| Hypertension exacerbated |
| Hypertension malignant |
| Hypertension NOS |
| Hypertension not adequately controlled |
| Hypertension paroxysmal |
| Hypertension rebound |
| Hypertension renal |
| Hypertension secondary |
| Hypertension secondary to renal disease, antepartum |
| Hypertension secondary to renal disease, comp preg, childbirth, and the puerp, unspec as to eoc |
| Hypertension secondary to renal disease, complicating pregnancy, childbirth, and the puerperium |
| Hypertension secondary to renal disease, complicating pregnancy, childbirth, and the puerperium, uns |
| Hypertension secondary to renal disease, postpartum |
| Hypertension secondary to renal disease, with delivery |
| Hypertension secondary to renal disease, with delivery, with mention of postpart comp |
| Hypertension secondary to renal disease, with delivery, with mention of postpartum complication |
| Hypertension worsened |
| Hypertensive |
| Hypertensive cardiomegaly |
| Hypertensive crisis |
| Hypertensive encephalopathy |
| Hypertensive episode |
| Hypertensive episodes |
| Hypertensive heart and renal disease |
| Hypertensive heart and renal disease NOS |
| Hypertensive heart and renal disease, benign |
| Hypertensive heart and renal disease, benign, with congestive heart failure |
| Hypertensive heart and renal disease, benign, with congestive heart failure and renal failure |
| Hypertensive heart and renal disease, benign, with renal failure |
| Hypertensive heart and renal disease, benign, without mention of congestive heart or renal failure |
| Hypertensive heart and renal disease, malig, w/o ment of congestive heart failure or renal failure |
| Hypertensive heart and renal disease, malignant |
| Hypertensive heart and renal disease, malignant, with congestive heart failure |
| Hypertensive heart and renal disease, malignant, with congestive heart failure and renal failure |
| Hypertensive heart and renal disease, malignant, with renal failure |
| Hypertensive heart and renal disease, unspec, w/o ment of congestive heart failure or renal failure |
| Hypertensive heart and renal disease, unspecified |
| Hypertensive heart and renal disease, unspecified, with congestive heart failure |
| Hypertensive heart and renal disease, unspecified, with congestive heart failure and renal failure |
| Hypertensive heart and renal disease, unspecified, with renal failure |
| Hypertensive heart disease |
| Hypertensive heart disease NOS |
| Hypertensive heart disease, benign |
| Hypertensive heart disease, malignant |
| Hypertensive heart disease, unspecified |
| Hypertensive renal disease |
| Hypertensive renal disease NOS |
| Hypertensive renal disease, benign |
| Hypertensive renal disease, benign, with renal failure |
| Hypertensive renal disease, benign, without mention of renal failure |
| Hypertensive renal disease, malignant |
| Hypertensive renal disease, malignant, with renal failure |
| Hypertensive renal disease, malignant, without mention of renal failure |
| Hypertensive renal disease, unspecified |
| Hypertensive renal disease, unspecified, with renal failure |
| Hypertensive renal disease, unspecified, without mention of renal failure |
| Hypertensive retinopathy |
| Hypertensive retinopathy grade 3 |
| Hypertensive retinopathy grade 4 |
| Hypertensive retinopathy grades 1 and 2 |
| Increased blood pressure |
| Increased BP slipping out of control |
| Insulin resistance syndrome |
| Labile blood pressure |
| Malignant essential hypertension |
| Malignant hypertension |
| Malignant hypertension NOS |
| Malignant hypertensive heart and renal disease |
| Malignant hypertensive heart disease |
| Malignant hypertensive heart disease with congestive heart failure |
| Malignant hypertensive heart disease without congestive heart failure |
| Malignant renal hypertension |
| Malignant renovascular hypertension |
| Malignant secondary hypertension |
| Malignant secondary hypertension NOS |
| MAP increased |
| Maternal hypertension affecting foetus |
| Maternal hypertensive disorders affecting fetus or newborn |
| Mean arterial pressure high |
| Mean arterial pressure increased |
| Metamephrine urinary elevated |
| Metanephrine urine abnormal NOS |
| Metanephrine urine increased |
| Mild or unspecified pre-eclampsia |
| Mild or unspecified pre-eclampsia, antepartum |
| Mild or unspecified pre-eclampsia, postpartum |
| Mild or unspecified pre-eclampsia, unspecified as to episode of care |
| Mild or unspecified pre-eclampsia, with delivery |
| Mild or unspecified pre-eclampsia, with delivery, with mention of postpartum complication |
| Nephropathy hypertensive |
| Norepinephrine abnormal NOS |
| Norepinephrine elevated serum |
| Norepinephrine increased |
| Other benign secondary hypertension |
| Other malignant secondary hypertension |
| Other pre-existing hypertension complicating pregnancy, childbirth, and the puerperium |
| Other pre-existing hypertension complicating pregnancy, childbirth, and the puerperium, unspecified |
| Other pre-existing hypertension, antepartum |
| Other pre-existing hypertension, postpartum |
| Other pre-existing hypertension, with delivery |
| Other pre-existing hypertension, with delivery, with mention of postpartum complication |
| Other unspecified secondary hypertension |
| PIH Pregnancy induced hypertension |
| Plasma aldosterone abnormal |
| Plasma aldosterone increased |
| Plasma catecholamines abnormal |
| Plasma catecholamines increased |
| Pre-eclamp/ eclamp superimposed on pre-existing hyperten, with delivery, with ment of postpart comp |
| Pre-eclamp/eclamp superimp on preexist hyperten, comp preg, c/birth or the puerp, unspec as to eoc |
| Pre-eclampsia |
| Pre-eclampsia or eclampsia superimposed on pre-existing hypertension |
| Pre-eclampsia or eclampsia superimposed on pre-existing hypertension, antepartum |
| Pre-eclampsia or eclampsia superimposed on pre-existing hypertension, complicating pregnancy, childb |
| Pre-eclampsia or eclampsia superimposed on pre-existing hypertension, postpartum |
| Pre-eclampsia or eclampsia superimposed on pre-existing hypertension, with delivery |
| Pre-eclampsia or eclampsia superimposed on pre-existing hypertension, with delivery, with mention of |
| Pre-eclampsia toxemia |
| Pre-eclamptic toxaemia |
| Pregnancy induced hypertension |
| Pressure arterial increased |
| Pressure blood increased |
| Primary hyperaldosteronism |
| Primary hypertension |
| Pseudo aldosteronism |
| Pseudoaldosteronism |
| Raised blood pressure |
| Raised BP |
| Rebound hypertension |
| Refractory hypertension |
| Renal hypertension |
| Renal hypertension NOS |
| Renin abnormal |
| Renin high |
| Renin increased |
| Renovascular hypertension |
| Retinopathy hypertensive |
| Retinopathy hypertensive grade 3 |
| Retinopathy hypertensive grade 4 |
| Retinopathy hypertensive grades 1 and 2 |
| Rise in blood pressure |
| Rise in BP |
| SBP increased |
| Secondary aldosteronism |
| Secondary hypertension |
| Secondary hypertension (non-renal) |
| Secondary hypertension NOS |
| Secondary hypertension, benign |
| Secondary hypertension, malignant |
| Secondary hypertension, unspecified |
| Serum aldosterone abnormal |
| Serum aldosterone increased |
| Serum catecholamines abnormal |
| Serum catecholamines increased |
| Serum noradrenaline increased |
| Severe pre-eclampsia |
| Severe pre-eclampsia, antepartum |
| Severe pre-eclampsia, postpartum |
| Severe pre-eclampsia, unspecified as to episode of care |
| Severe pre-eclampsia, with delivery |
| Severe pre-eclampsia, with delivery, with mention of postpartum complication |
| Systolic hypertension |
| Systolic pressure increased |
| Toxaemia of pregnancy |
| Toxemia of pregnancy |
| Transient hypertension of pregnancy |
| Transient hypertension of pregnancy, antepartum |
| Transient hypertension of pregnancy, postpartum |
| Transient hypertension of pregnancy, unspecified as to episode of care |
| Transient hypertension of pregnancy, with delivery |
| Transient hypertension of pregnancy, with delivery, with mention of postpartum complication |
| Uncomplicated hypertension |
| Unspec hypertension comp preg,childbirth, or the puerperium, unspec as to eoc |
| Unspecified antepartum hypertension |
| Unspecified essential hypertension |
| Unspecified hypertension complicating pregnancy, childbirth, or the puerperium |
| Unspecified hypertension complicating pregnancy, childbirth, or the puerperium, unspecified as to ep |
| Unspecified hypertension, with delivery |
| Unspecified hypertension, with delivery, with mention of postpartum complication |
| Unspecified hypertensive heart disease |
| Unspecified hypertensive heart disease with congestive heart failure |
| Unspecified hypertensive heart disease without congestive heart failure |
| Unspecified postpartum hypertension |
| Unspecified renovascular hypertension |
| Unspecified secondary hypertension |
| Urinary aldosterone increased |
| Urinary catecholamines elevated |
| Urinary metanephrine increased |
| Withdrawal hypertension |
| Pre-eclampsia aggravated |
| HELLP syndrome |
| Labile hypertension |
| Normetanephrine urine increased |
| Renin-angiotensin system inhibition |
| Angiotensin converting enzyme increased |
| Renal parenchymal hypertension |
| Hypertension neonatal |
| Hypertension on emergence |
| Keith-Wagener-Barker retinopathy grade 1 |
| Keith-Wagener-Barker retinopathy grade 2 |
| Keith-Wagener-Barker retinopathy grade 3 |
| Keith-Wagener-Barker retinopathy grade 4 |
| Hypertension retinopathy grade 1 |
| Hypertension retinopathy grade 2 |
| Isolated systolic hypertension |
| Postoperative hypertension |
| Blood pressure inadequately controlled |
| White coat hypertension |
| Metabolic syndrome |
| Metabolic syndrome X |
| Diuretic therapy |
| Renal revascularisation surgery |
| Blood pressure orthostatic abnormal |
| Blood pressure orthostatic increased |
| Hypertensive nephropathy |
| Renal revascularization surgery |
| Borderline hypertension |
| Endocrine hypertension |
| Hypertensive emergency |
| Hypertensive urgency |
| Hypertensive cardiomyopathy |
| Hypertensive angiopathy |
| Maternal hypertension affecting fetus |
| Catecholamines urine increased |
| Aldosterone urine abnormal |
| Catecholamines urine abnormal |
| Epinephrine abnormal |
| Norepinephrine abnormal |
| Metanephrine urine abnormal |
| Dysmetabolic syndrome |
| Gestosis |
| Fundus hypertonicus |
| Procedural hypertension |
| Intraoperative hypertension |
| Dialysis induced hypertension |
| Blood pressure management |
| Non-dipping |
| Absence of nocturnal arterial blood pressure descent |
| Orthostatic hypertension |
| Prehypertension |
| Uncontrolled hypertension |
| Blood pressure reading high |
| Neurogenic hypertension |
| Tyramine reaction |
| Gestational hypertension |
| Renal artery ablation |
| Renal artery denervation |
| Pre-eclampsia toxaemia |
| Transient blood pressure increase |
| Renal sympathetic nerve ablation |
| Hyperaldosteronemia |
| Hyperaldosteronaemia |
| Page kidney |
| Hypertensive cerebrovascular disease |
| Hypertensive angiosclerosis |
| Supine hypertension |
| Supine diastolic blood pressure increased |
| Hypertensive end-organ damage |
| Angiotensin II receptor type 1 antibody positive |
| Renal compression |
| Resistant hypertension |
| Arterial hypertension |
| Catecholamine crisis |
| Cardiometabolic syndrome |
| Severe asymptomatic hypertension |
| Copper wiring |
| Silver wiring |
| Renal vascular resistance increased |
| Pseudohyperaldosteronism |
| Superimposed pre-eclampsia |
| Hypertension during dialysis |
| Controlled hypertension |
| Pre-eclampsia with severe features |
| Angiotensin converting enzyme abnormal |
| Renal artery revascularization |
| Renal artery revascularisation |
| Systemic hypertension |
| Syndrome Z |
| Preeclampsia |
| Nocturnal hypertension |
| Reverse dipping |
| White coat syndrome |
| Metabolic inflammation |
| Pediatric hypertension |
| Paediatric hypertension |
| Juvenile hypertension |
| Postpartum pre-eclampsia |
