# Supplementary material for: Hypertension associated with serotonin reuptake inhibitors: A new analysis in the WHO pharmacovigilance database and examination of dose-dependency
Source: PLoS One. 2025 Mar 7;20(3):e0317841. doi: 10.1371/journal.pone.0317841 (PMC11888134; doi:10.1371/journal.pone.0317841)
Supplement: Attachment — Submitted filename: reply_v3.docx [file pone.0317841.s004.docx]

PONE-D-24-22756 – R1: Hypertension associated with serotonin reuptake inhibitors: an updated analysis in the WHO pharmacovigilance database and examination of dose-dependency

**Reviewer #1:**

Thank you for the opportunity given to review this work. I appreciate the study conducted by the authors as the findings bring new insight to the specific topic. Please find below some suggestions to add to the quality of this paper regarding clarification.

**REPLY: We appreciate the straightforward synthetized message that this Expert. We thank her/him for the insightful comments raised. In the revised MS all changes introduced are outlined in yellow. Pages and lines indicated in brackets at the end of our comments in this document are taken from the clean manuscript.** **Finally, we updated the data from our study.**

TITLE/ABSTRACT
Comment 1: the word “an updated” seems a bit ambiguous without clear information given.

**REPLY: Thank you for this important message. We propose this new title : “Hypertension associated with serotonin reuptake inhibitors: a new analysis in the WHO pharmacovigilance database and examination of dose-dependency” (page 1, line 1).**

Comment 2: “..predominantly in women (69.9%) within the age range of 45-64 years (45.2%).” Is the proportion of 45.2% for females only?. It seems there are two different information that should not be combined in this sentence.

**REPLY: Thank you for this important message. We have rephrased this sentence (page 2, line 44).**

MATERIALS AND METHODS/RESULTS/DISCUSSION

Comment 3: “Hypertension (broad)” definition based on MedDRA SOC is used by the authors to identify cases of interest. Does this selection include gestational hypertension cases as well?. Additional information is suggested to clarify this.

**REPLY: Thank you for this important message. We have precisely described “Hypertension (broad)” definition based on MedDRA SOC in** **Supplementary Table III and this definition includes gestational hypertension. We have precised this point in the Method section (page 7, lines 125-126).**

Comment 4: Did the authors consider the positive dechallenge or rechallenge given in the reports when selecting hypertensive cases?. Is there a preference for drug characterisation by reporters (for example only reports where an SRI drug is suspected) when including the cases for the analyses?

**REPLY: Thank you for this important message. Positive dechallenge and rechallenge are now described in Table V and a reminder is given in Results section (page 9, line 159). Moreover, All SRIs reported, regardless of their status (suspect, concomitant or interaction), were included in the analyses, as is it standard practice in pharmacoepidemiological analyses. We have precised this point in the Method section (page 7, lines 127-128).**

Comment 5: There are also other drugs reported to (possibly) induce hypertension (<https://doi.org/10.1016/j.ecl.2019.08.013>). Supposedly these drugs are also mentioned in the reports together with SRI drugs of interest, the chance for hypertension may increase because of potential drug interactions. Did the authors take this possibility into account?

**REPLY: Thank you for this important message. Now, in the multivariate analysis, drugs known to cause hypertension have been included in the model (from ATC codes) in addition to age category, sex, antihypertensive drugs. This point is precised in Method section (page 8, lines 137-138).**

Comment 6: Could the authors add additional information if there is a plausible overlapping between the timing use of an SRI and the onset of hypertension mentioned in the reports?

**REPLY: Thank you for this important message. Now, Table IV summarises, for each SRI, the time to onset of hypertension after their introduction, using the median and interquartile ranges. We have precised this point in the Method section (page 8, lines 141-142).**

**Reviewer #2:**

The authors tackle a question that is still unresolved in pharmacovigilance: the risk of hypertension (HTN) in patients treated with SSRIs. They rely on a classic but well-established methodology of disproportionality, partially multivariate, and have rightly followed the recent READUS-PV guidelines.

**REPLY: We appreciate the straightforward synthetized message of this Expert. We thank her/him for the insightful comments raised. In the revised MS all changes introduced are outlined in yellow. Pages and lines indicated in brackets at the end of our comments in this document are taken from the clean manuscript. Finally, we updated the data from our study**

Methodologically, it seems necessary to clarify the inclusion criteria for cases. Based on the numbers provided by the authors, it appears that they have chosen to include all cases mentioning an SSRI (suspect OR concomitant), not just the cases where the SSRI was considered suspected. This choice is not necessarily absurd, but it must be clearly stated in the methodology.

**REPLY: Thank you for this important message. All SRIs reported, regardless of their status (suspect, concomitant or interaction), were included in the analyses. We have precised this point in the Method section (page 7, lines 127-128).**

The analysis would benefit from a sensitivity analysis restricted to cases where SSRIs are suspected (excluding concomitant cases). I’m not sure that disproportionality remains positive with these criteria, according to the tests I conducted on the database.

**REPLY: Thank you for this important message. We have conduced sensitivity analysis as advised by reviewer. These results are now in Results section (page 9, lines 171-173) and Supplementary Table I.**

To strengthen their results, the authors might consider comparing the effect of SSRIs on blood pressure with that produced by other medications. It would be very interesting to compare SSRIs to another medication used in a similar population (depressed patients) to mitigate some of the confounding biases. However, the challenge is that most antidepressants are more or less strongly associated with the occurrence of HTN (tricyclics, MAO inhibitors, SNRIs…). As a fallback, perhaps a comparison of SSRIs vs all other reported antidepressants and/or SSRIs vs SNRIs and/or SSRIs vs mirtazapine/mianserine could be proposed. This would better account for the ROR.

**REPLY: Thank you for this important message. We have conduced sensitivity analysis as advised by reviewer. These results are now in Results section (pages 9-10 , lines 173-176) and Supplementary Table II.**

The results section is somewhat brief, even though it refers to high-quality tables. It could potentially be slightly expanded to balance the article.

**REPLY: Thank you for this important message. We have expanded the Results section to include the results of sensitivity analyses (pages 9-10, lines 171-176).**

The scientific literature is relatively rich in studies that contribute to the debate on the HTN-SSRI link. This debate might warrant a more precise discussion in the article. In particular:

- Reference 7 on tricyclics only shows a trend toward grade 1 HTN (lower bound of the OR <1), which should be clarified in the introduction.

**REPLY: Thank you for this important message. We have precised this point in Introduction section (page 5, lines 90-91).**

- The following article deserves to be discussed (in the introduction or discussion), especially since it seems rather reassuring regarding the effect of SSRIs on blood pressure (with even a decrease in diastolic pressure observed): <https://doi.org/10.1016/j.jad.2023.08.032>

**REPLY: Thank you for this important message. We have precised this point in Discussion section (page 11, lines 197-201).**

- The following article could also be mentioned, even if it is more consistent with the authors’ theory in the manuscript: <https://doi.org/10.1016/j.psychres.2023.115300>

**REPLY: Thank you for this important message. We have precised this point in Discussion section (pages 10-11, lines 192-197).**

- From a pathophysiological perspective, the hypothesis by Montastruc et al. regarding the NET/SERT ratio is interesting to mention, even if it leans more toward the determining role of NET compared to the serotonergic effect.

**REPLY: Thank you for this important message. We have precised this point in Discussion section (page 12, lines 217-219).**

One minor point: it seems that there is a missing arrow in Fig 1 (flowchart).

**REPLY: Thank you for this message.** **It has been added.**

Overall, this is a very interesting article that deserves some adjustments to become fully convincing.

**REPLY: Thank you for your help in improving the quality of our article.**
